# Pseudo-Outbreak of *Purpureocillium lilacinum* Skin Infections at a Dermatology Clinic — Washington, 2024

**DOI:** 10.15585/mmwr.mm7505a2

**Published:** 2026-02-12

**Authors:** Kyle S. Yomogida, Emily Laskowski, Lisa Hannah, Elizabeth Sajewski, Paige Gable, Heather Moulton-Meissner, Kristen Clancy, Marissa Howard, Mitsuru Toda, Anastasia P. Litvintseva, Lindsay A. Parnell, Brian Schwem, Kimberly L. McKinney, Amy L. Valderrama, Kiran M. Perkins, Samia N. Naccache, Dallas J. Smith

**Affiliations:** ^1^Washington State Department of Health; ^2^Epidemic Intelligence Service, CDC; ^3^Council of State and Territorial Epidemiologists, Atlanta, Georgia; ^4^Mycotic Diseases Branch, Division of Foodborne, Waterborne, and Environmental Diseases, CDC; ^5^Division of Healthcare Quality Promotion, National Center for Emerging and Zoonotic Infectious Diseases, CDC; ^6^Labcorp, Seattle, Washington.

**Summary**

**What is already known about this topic?**

*Purpureocillium lilacinum* is a common environmental mold. *P. lilacinum* skin infections are rare and mostly occur in immunocompromised persons.

**What is added by this report?**

In 2024, a Washington dermatology clinic that collected skin swab specimens for diagnosis of fungal skin infections, rather than the recommended biopsy or skin scraping specimens, reported 22 patients with cultures yielding *P. lilacinum.* All swab specimens were contaminated by saline from squeeze bottles that had been repeatedly refilled and reused by clinic staff members during specimen collection. Isolates from patient swab specimens and from the contaminated bottles were closely related by whole genome sequencing; in this investigation, no patient received confirmation of clinical infection with *P. lilacinum*.

**What are the implications for public health practice?**

Proper specimen collection procedures, diagnostic practices, and infection prevention and control measures are critical to providing recommended care in clinical settings.

## Abstract

In April 2024, a Washington dermatology clinic reported multiple cases of atypical fungal skin infections caused by the environmental mold *Purpureocillium lilacinum* to Clallam County Public Health. *P. lilacinum* most frequently causes skin infections among immunocompromised persons but can occasionally infect immunocompetent persons. The diagnoses were made on the basis of swab specimens collected from patients’ skin to diagnose a fungal skin infection, rather than from a biopsy or skin scraping, as is recommended. An investigation by the Washington State Department of Health and CDC revealed this to be a pseudo-outbreak caused by contaminated saline, ultimately affecting 22 patients during January–October 2024. The investigation found that the clinic had refilled and reused saline squeeze bottles during skin swab collection. This resulted in contamination of the saline with *P. lilacinum*, which was transferred to the skin when swab specimens were obtained. Whole genome sequencing confirmed that *P. lilacinum* isolates from patient swab specimens were closely related to environmental isolates from the contaminated bottles. The clinic discontinued use of refillable squeeze bottles in January 2025 and transitioned to single-use saline packets, after which no further cases were reported. This investigation underscores the importance of proper diagnostic specimen collection procedures and infection prevention and control practices in clinical settings.

## Investigation and Findings

### Public Health Notification

In April 2024, a Washington dermatology clinic notified Clallam County Public Health of atypical fungal skin infections in six patients who had been evaluated at the clinic during January 4–April 1, 2024. The patients reported rashes of varying descriptions, with onsets ranging from weeks to months before seeking care at the clinic, and all six had *Purpureocillium lilacinum* cultured from skin swabs. By June 2024, the clinic had identified *P. lilacinum* in skin swabs collected from 15 patients. However, because fungal skin infections can only be confirmed through punch biopsy or skin scrapings (*1*), the Washington State Department of Health (DOH) and CDC initiated an investigation into the reported cluster to verify the diagnoses and determine whether a common exposure existed among patients with *P. lilacinum* cultures from skin swabs reported by the clinic. This investigation was reviewed by CDC, deemed not research, and was conducted consistent with applicable federal law and CDC policy.*

### Epidemiologic Investigation

**Patient record review.** DOH abstracted medical records and interviewed 15 patients reported with *P. lilacinum* cultured from their clinical specimens as of June 2024. Records included information from the index visit, medical history, and most recent progress notes. Abstracted information included demographic characteristics, *P. lilacinum* laboratory testing results, and clinical history (e.g., signs, symptoms, preexisting conditions, surgeries, and treatment). Because little was known about potential sources of infection, telephone interviews using a standardized questionnaire inquired about a wide range of potential community exposures, including animals, recreational activities (e.g., use of recreational water sources and outdoor activities), social spaces (e.g., gyms and social organization or group settings), congregate living settings, outdoor occupations, and skin care products applied to the rash site.

**Clinical characteristics.** Among 15 patients with reported positive *P. lilacinum* culture results during January 4–June 11, 2024, all but one had a rash; the reason the patient without a rash received testing is not known. Among the 14 patients with rashes, the rash was on the lower extremities of eight patients, on the head or neck of four patients, and in the genital area of two patients. Only one patient had had surgery during the year preceding rash onset, and the surgical site differed from the rash location. Among all 15 patients, 10 were prescribed topical antifungal medications, including seven who received ketoconazole and three who received clotrimazole. No information about the reported effectiveness of these medications in resolving reported rashes among patients is available.

**Ascertainment of potential exposures.** DOH completed interviews with 10 patients, eight of whom reported exposures to pets or participation in yardwork or gardening. Three of 10 patients reported frequenting social spaces, and two patients reported visiting outdoor recreation spaces or water sources, having an outdoor occupation, or living in a congregate setting. Six of the 10 patients reported using various ointments, lotions, or other skin care products near the rash site before receiving testing for *P. lilacinum.* Neither a common product used by all patients nor a common community exposure source was identified.

**Review of fungal culture results and identification of additional cases.** On October 9, 2024, the clinic reported to DOH seven additional patients with *P. lilacinum* isolates from specimens collected during July 22–September 30, 2024, for a total of 22 patients in 2024. Fungal cultures yielding *P. lilacinum* performed by a commercial laboratory (Labcorp) during January 2023–October 2024 were analyzed. The laboratory, which received specimens from the clinic, reported that the number of *P. lilacinum* isolates from Washington during 2024 (30) was approximately four times the number reported during 2023 (seven) and that 28 (93%) of 2024 Washington specimens were skin swabs submitted by the clinic in 2024. The laboratory did not observe an increase in *P. lilacinum* isolates from other states during this period. DOH contacted other dermatology clinics in the region and did not identify any additional *P. lilacinum* isolates. The clinic did not provide additional patient line lists after October 9, 2024; thus, DOH was not able to reconcile the number of isolates reported by the laboratory with the number of patients examined at the clinic or to verify that the 30 laboratory test results represented unique patients.

**Review of clinic procedures.** On January 13, 2025, DOH conducted a telephone consultation with the clinic to discuss its specimen collection protocol. The clinic reported that, when swabbing dry, nonpurulent rashes of unknown etiology, the clinic health care provider would first place a drop of saline solution from a refillable squeeze bottle onto sterile gauze pad and wipe the skin where the rash was located (Figure). A drop of saline from the same squeeze bottle would then be placed onto the tip of the sterile swab before swabbing the rash and packing the swab in the transport tube. The plastic squeeze bottles were repeatedly opened and refilled using commercially produced single-use, 1-L containers of 0.9% sodium chloride (sterile saline) solution. The same squeeze bottles had been used regularly by clinic providers for at least 1 year and were not cleaned or sterilized between refills. Leftover solution from 1-L saline containers was occasionally saved and used to refill the squeeze bottles as needed. DOH observed this process during a subsequent on-site visit in February 2025, which included a specimen collection procedure demonstration.

**FIGURE:**
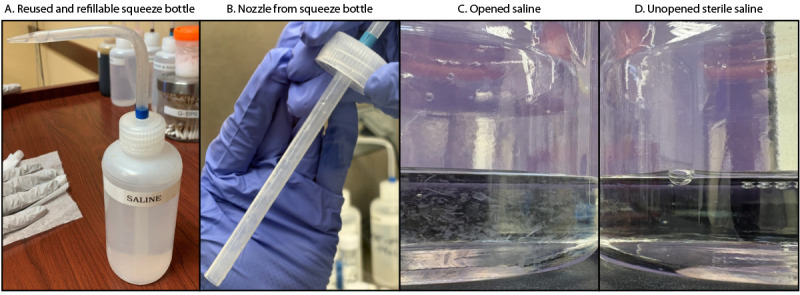
Refillable squeeze bottle contaminated with *Purpureocillium lilacinum* (A), cap and nozzle from refillable squeeze bottle (B), and opened (C) and unopened (D) saline bottles* used to refill squeeze bottles — Washington, 2024 Photos/Washington State Department of Health; CDC * Opened bottle includes particulate matter; unopened (sterile) bottle is clear.

### Laboratory Investigation

In February 2025, DOH visited the clinic and retrieved six refilled squeeze bottles, four 1-L source saline bottles (three opened and one unopened), and three unopened swabs. An additional swab, used by clinic staff members to demonstrate specimen collection with single-use saline during the DOH visit, was also collected. All samples were received by CDC for culture and identification of *P. lilacinum*. Aliquots (10-mL and 100-mL samples from the squeeze bottles and the source saline bottles, respectively) were filtered through 0.22-micron filters and cultured on Sabouraud dextrose agar with chloramphenicol and gentamicin (SAB with C+G). Swab samples were extracted in phosphate-buffered saline containing 0.02% Tween-80, vortexed, and plated on SAB with C+G. Plates were incubated aerobically at 98.6°F (37°C) and observed for up to 7 days for growth of suspected *P. lilacinum* colonies. Confirmatory identification of suspected *P. lilacinum* isolates was performed on the basis of polymerase chain reaction (PCR) amplification and Sanger sequencing of the internal transcribed spacer (ITS) region of the ribosomal RNA (rRNA) gene locus within the ribosomal DNA that codes for rRNA using ITS4 and five PCR primers (*2*,*3*). *P. lilacinum* was identified from the six refilled squeeze bottles and one of the 1-L opened source saline bottles. *P. lilacinum* was not isolated from the unopened 1-L source saline bottles, the demonstration swab used by clinic staff, or any of the unopened swabs. In addition to these tests, one isolate from a patient’s skin swab was sent from the commercial laboratory to CDC; whole genome sequencing was performed to assess relatedness among this skin swab isolate, environmental isolates, and nonrelated comparator isolates. The skin swab isolate and environmental isolates were within 100 single nucleotide polymorphisms (SNPs) of each other; comparator isolates differed by approximately 200,000 SNPs.

## Public Health Response

On January 28, 2025, DOH provided clinical standards of care information to the clinic and advised changing specimen collection procedures. DOH advised the clinic to immediately remove all reusable squeeze bottles in the clinic. In addition, DOH advised the clinic to confirm suspected fungal skin infections through biopsies, not superficial skin swabs. On January 29, 2025, clinic staff members transitioned to using single-use saline packets. Since February 2025, no swabs have been submitted for fungal cultures to the commercial laboratory and no patients with cultures yielding *P. lilacinum* have been reported by the clinic. During a telephone check-in with the clinic on May 29, 2025, clinic staff members noted that none of the patients who had *P. lilacinum* erroneously diagnosed by skin swab have had subsequent biopsies or other clinical specimens collected. DOH advised clinic staff members to reevaluate diagnoses of patients who had *P. lilacinum* isolates identified from cultures. As of June 5, 2025, none of the patients had received an alternative diagnosis. No evidence indicates that *P. lilacinum* caused the reported rashes among patients. 

## Discussion

*P. lilacinum* is a mold found commonly in the environment. Skin infections caused by *P. lilacinum* most frequently occur among immunocompromised persons (*4*). Previous reports of *P. lilacinum*–associated skin infections (*4*–*6*) describe tender, red plaques with pustules or ulcers. Many reported *P. lilacinum* infections have been associated with having had a recent surgical procedure or having an immunocompromising condition (*7*). Other clinical signs of *P. lilacinum* infections include invasive disease, endocarditis, fungal keratitis, and pneumonia (*8*,*9*).

In this investigation of a *P. lilacinum* pseudo-outbreak, no patient had undergone recent surgery or was immunocompromised. No patient received confirmation of a clinical infection through *P. lilacinum* detected from a biopsy specimen or skin scraping or had a rash consistent with a *P. lilacinum* skin infection. In addition, whole genome sequencing indicated that *P. lilacinum* isolated from a patient’s skin swab was closely related to environmental isolates (e.g., refilled saline squeeze bottles), providing strong evidence that swabs were contaminated at the time of specimen collection. Because the date when the squeeze bottles were first used in the clinic is not known, the mechanism that led to fungal colonization of the squeeze bottles cannot be determined. Fungi were likely present in or near the clinic and proliferated within multiple squeeze bottles because they were being refilled without sterilization.

The identification of this pseudo-outbreak depended on close collaboration among the commercial laboratory, state and local public health agencies, and CDC. Coordination among all parties enabled timely data sharing and resulted in the identification of the contamination source.

### Implications for Public Health Practice

This investigation highlights the value of using recommended diagnostic procedures and implementation of infection prevention and control procedures to avert contamination of medical equipment. An appropriate tissue specimen (e.g., punch biopsy) is needed to confirm a fungal skin infection. 

Refilling bottles with aqueous solutions for clinical use can lead to proliferation of certain microorganisms (*10*). Although this pseudo-outbreak did not involve clinical skin infections caused by *P. lilacinum*, exposing an open wound to contaminated products or medical equipment could result in infection. Antifungal treatment should only be prescribed when appropriate clinical specimens (e.g., biopsy) confirm clinical infection. To reduce the risk for misdiagnosis due to clinical specimen contamination, medical equipment and products must be used and maintained according to manufacturers’ instructions.

## Footnotes

* 45 C.F.R. part 46, 21 C.F.R. part 56; 42 U.S.C. Sect. 241(d); 5 U.S.C. Sect. 552a; 44 U.S.C. Sect. 3501 et seq.
